# Population-Level Data on Child Development at School Entry Reflecting Social Determinants of Health: A Narrative Review of Studies Using the Early Development Instrument

**DOI:** 10.3390/ijerph18073397

**Published:** 2021-03-25

**Authors:** Magdalena Janus, Caroline Reid-Westoby, Noam Raiter, Barry Forer, Martin Guhn

**Affiliations:** 1 Offord Centre for Child Studies, Department of Psychiatry and Behavioural Neurosciences, McMaster University, Hamilton, ON L8S 4L8, Canada; reidwc@mcmaster.ca; 2 Human Early Learning Partnership, School of Population and Public Health, University of British Columbia, Vancouver, BC V6T 1Z3, Canada; barry.forer@ubc.ca (B.F.); martin.guhn@ubc.ca (M.G.); 3 Michael G. DeGroote School of Medicine, McMaster University, Hamilton, ON L8P 1H6, Canada; noam.raiter@gmail.com

**Keywords:** Early Development Instrument, developmental health, social determinants of health

## Abstract

Background: The Early Development Instrument (EDI) was developed as a population-level assessment of children’s developmental health at school entry. EDI data collection has created unprecedented opportunities for population-level studies on children’s developmental outcomes. The goal of this narrative review was to synthesize research using the EDI to describe how it contributes to expanding the understanding of the impacts of social determinants on child development and how it applies to special populations. Methods: Select studies published in peer-reviewed scientific journals between 2015 and 2020 and incorporating the social determinants of health perspectives were chosen to highlight the capability of the EDI to monitor children’s developmental health and contribute knowledge in the area of early childhood development. Results: A number of studies have examined the association between several social determinants of health and children’s developmental outcomes, including hard-to-reach and low-frequency populations of children. The EDI has also been used to evaluate programs and interventions in different countries. Conclusions: The ability of the EDI to monitor children’s developmental outcomes in various populations has been consistently demonstrated. The EDI, by virtue of its comprehensive breadth and census-like collection, widens the scope of research relating to early childhood development and its social determinants of health.

## 1. Introduction

Over 20 years ago, Dan Keating and Clyde Hertzman formulated a framework connecting early child development with the wealth and health of nations [1], introducing the term “developmental health.” This term was created to emphasize the intersection between different aspects of health, operationalizing the World Health Organization’s definition of health, described as more than just the absence of illness. The idea is that health includes components of physical, mental, and social well-being, which are linked and intertwined, whereby improvements in abilities in one area require the promotion and support of other areas [2]. It is now widely recognized that developmental health extends beyond cognitive abilities and combines children’s physical, mental, social, and emotional well-being [1,3]. Until early in the 21st century, much of the research on child development at school entry focused on cognitive abilities or a concept of school readiness that rarely went beyond the academic aspects [4]. Longitudinal studies show that measuring children’s cognitive abilities leaves significant variance in later academic achievement unexplained [5]; other childhood characteristics—factors such as motivation, sociality, self-regulation, and physical capacities—influence success in school [6,7]. More importantly, however, the strongest predictors of school success are often social context factors, such as poverty, opportunities for learning, and environments in which children learn [8].

In the early 2000s, researchers began examining developmental health from a more holistic perspective. One tool developed in Canada in the late 1990s was designed to do just this—the Early Development Instrument (EDI) [9]. The EDI was developed as a population-level assessment of children’s developmental health at school entry, taking a developmental epidemiology approach [10,11], which is used to characterize the distribution of children’s developmental health in kindergarten children and examine factors that might be associated with their developmental vulnerabilities. Its implementation in jurisdictions across Canada and internationally has led to many population-level studies on children’s developmental outcomes, including the examination of the associations with social determinants of health. The population-level approach is achieved through assessment of all children in a jurisdiction; in the case of the EDI, data are collected for each child in kindergarten [12]. 

Through this approach, the EDI has enabled researchers to examine hard-to-reach, low-frequency populations of children. Representative research evidence on minority groups is needed to effectively plan and implement large-scale interventions to improve children’s developmental health. To create universal change and lower the burden of developmental vulnerability for all children, widespread monitoring using valid instruments and reliable reporting is required [13]. The EDI has been a valuable tool in providing empirical evidence on the status of kindergarten children’s developmental outcomes, something that has led to the implementation of various child-related policies. Before the development of the EDI, the majority of developmental research was sample-based. While sample-based studies are informative, they are unable to examine certain associations that population-level studies can, and thus are limited in terms of their ability to provide comprehensive answers [14,15]. In contrast, population-level studies allow for comprehensive representativeness, including subpopulations of children, such as minority groups, which tend to be much less represented in sample-based studies. 

The EDI has also enabled researchers to examine the impacts of early childhood programs and interventions meant to help improve developmental outcomes for children, for example by evaluating the effectiveness of preschools [16,17] or in-home interventions [18,19]. As the understanding of the pervasive influence of social determinants of health on children development increased, it has also become more evident that they may moderate the impacts of interventions [20]. Targeted interventions, such as Head Start in the USA for example [21], often by default address social determinants of health, as they focus on children in families experiencing poverty. In contrast, universal interventions, such as provision of preschool or full-day learning in kindergarten, are intended to reach everyone, but may have differential impacts depending on social determinants, such as neighborhood or family socioeconomic status [17,22].

In Canada, the EDI has been used for over 20 years, providing a population-wide view of early childhood development at school entry for over 1.2 million children. The EDI has been adapted and validated in many countries, including Australia, Ireland, Scotland, Sweden, Brazil, Chile, Estonia, Peru, Jordan, Mexico, and the United States [9]. Its utilization of teacher ratings makes the EDI a cost-effective way to gather population-level data and has allowed the collection of data from a variety of jurisdictions across the full spectrum of wealth and health. The EDI encompasses five developmental domains and provides a well-grounded, holistic view of early child development. 

Although the benefits of population-level data may seem obvious in theory, it is necessary to examine the extent to which population-level data on child development have indeed contributed to the research discourse relevant to implementation science and policy [14]. This article is part of a Special Issue entitled “Early Child Development: From Measurement to Optimal Functioning and Evidence-based Policy.” This article represents a narrative review of select policy-relevant studies that have involved an internationally widely used population-based tool for measurement of early child development outcomes—the EDI. In the United Nations Children’s Fund (UNICEF) Report Card #11 published in 2013 [23], the EDI was mentioned as being the only population-based tool that can be used to understand early child development outcomes in different national contexts—providing unique opportunities to study trends over time of childhood development outcomes, as well as variability in childhood development outcomes in connection with social context factors (e.g., poverty, access to resources, socioeconomic status).

In this article, we examine the ways in which research studies using population-based EDI measurement have been able to contribute to the discourse on “evidence-based policies” that seeks to enhance “optimal functioning” (e.g., positive health and education outcomes) of children. In the following, we will provide background information that situates the EDI within a context of linking international early child development research to social determinants of health and policy or decision-making that seeks to enhance population health. We then provide a narrative review of select studies using EDI data to address three questions related to the theme of the Special Issue and discuss remaing gaps and limitations, as well as future opportunties for population-based developmental health and social determinants of health (SDOH) research, in order to inform policy-making that enhances population health. In this narrative review, we synthesize selected published research on child development, conducted using the EDI as one of the measures. Our aim was to identify and describe the areas of research in which the EDI has:

(1) Widened the scope of understanding of the magnitude of the impacts of social determinants of health on child development by including large populations of children and hard-to-reach subgroups;
(2) Extended the understanding of how the social determinants of health impact developmental health in special populations of children;
(3) Contributed to the understanding of the impacts of early interventions on child development.

## 2. Materials and Methods 

This paper represents a “narrative review” [24], as we conducted a directed synthesis of select studies using population-level EDI data to illustrate the themes of this paper. In the following, we describe the study selection process and criteria for inclusion in this review.

### 2.1. Paper Selection Process

This qualitative overview started by looking at all peer-reviewed papers published between 2015 and 2020 that used EDI data as an outcome measure (included in the EDI Bibliography page, https://edi.offordcentre.com/resources/bibliography-of-the-edi/ (accessed on 23 February 2021). These papers (*n* = 133) were summarized based on the research question, population studied, analyses conducted, results, and new knowledge created. The summaries were then reviewed for suitability to the three research areas listed in the introduction. Papers had to describe an empirical study (either prospective or secondary data analysis), include research questions that could be categorized as addressing social determinants of health (including prevention or intervention programs), and identify the EDI as the main outcome measure. As a result, papers that represented study protocols or data repository profiles, straightforward validation studies, commentaries, or reviews were excluded. No restrictions were put on country or region of origin or sample size. The authors each selected five papers they considered as most relevant for each area of research and then agreed upon selection criteria for inclusion in this review via consensus. Once we reached a relative saturation level for a specific topic [25], we limited further inclusion of papers. During the process of writing the sections, the list of papers included in the review was expanded to incorporate papers published prior to 2015, resulting in an addition of two papers: one published in 2010 addressing intersectionality [26] and one published in 2013 [27], including data from Scotland to increase geographical coverage. Where possible, for each category, we included work that addressed diverse populations, represented several geographic regions, and was authored by researchers from a range of institutions. The findings of the 33 included papers are described and summarized in the results (13, 13, and 7 papers in each of the three areas of research, respectively). The limitations of this approach are addressed in the Discussion. 

### 2.2. Measures

#### Early Development Instrument

The EDI [9] is a population-level measure of children’s developmental health at school entry. It is a teacher-completed questionnaire that assesses children’s age-appropriate abilities in five different areas of development: physical health and well-being, social competence, emotional maturity, language and cognitive development, and communication skills and general knowledge. While teachers complete the EDI for every child in their class, the results are never interpreted at the individual level. Rather, they are aggregated and analyzed for groups of children (e.g., school, neighborhood, sex). For example, reports are provided to school authorities at the school- and district-level, to communities at the municipality-level, and to provinces or territories at the jurisdictional level. For research purposes, children are often grouped into various categories of interest (e.g., sex, age, illness, special needs, immigrant status) and results are compared between groups [9].

The EDI’s validity, reliability, and consistency has been extensively tested in a number of countries. In Canada, the EDI has shown internal consistency values in the range of 0.84 to 0.94 for the various domains, while an assessment of test–retest reliability showed values in the range of 0.80 to 0.90 [9]. Additionally, international studies have reported similar values of internal validity and test–retest reliability. A comparison across Canada, Australia, Jamaica, and the United States showed internal consistency values ranging between 0.62 to 0.94 [28]. Evidence of predictive validity has been provided by studies from Canada and Australia [6,29,30]. Additionally, studies have shown that EDI teacher ratings align well with those of parents and with other forms of developmental assessments [31,32,33]. Moreover, developmental vulnerability indicated by any of the five EDI domains in kindergarten is predictive of academic, emotional, and social incompetence in later elementary school years in Canada [30], Australia [29], and the USA [34].

## 3. Results

### 3.1. Social Determinants of Health and Early Childhood Development

It was evident early in the history of published EDI literature that it provided a new and useful vehicle for widening the scope of research on the effects of SDOH on children’s development, fulfilling and expanding the promise of such data predicted by Keating and Hertzman [1]. Indeed, by 2007, when the first peer-reviewed paper was published on the development and psychometric properties of the EDI [9], there were already seven papers published on the relationship between neighborhood-level EDI scores and their associated socioeconomic and demographic contexts. Most of these appeared in the first EDI-focused Special Issue in the journal “Early Education and Development” [32,35,36,37].

EDI literature examining SDOH contexts has demonstrated a steady output over time of over 60 articles, with at least five published in any two-year period going back to 2007, likely because of the EDI’s usefulness in studying the social determinants of health (SDOH) from a population perspective. In contrast, the majority of peer-reviewed literature on EDI psychometric properties was published in 2011 or earlier. 

Population-level studies, such as those made possible by the EDI, help to illustrate the fundamental and enduring impacts of SDOH on children’s health. Population-level studies commonly focus on modeling the effects of SDOH as the key variables of interest [38]. In psychological child development studies, it is common to relegate SDOH variables, such as parental education and household income, to the role of controlling for selection effects. When SDOH variables are explicitly modeled, studies have shown that they have stronger effects on children’s outcomes than child-level “risk factors”. For example, using population-level data in Manitoba, Brownell et al. found that family risk factors (e.g., being on income assistance) and neighborhood socioeconomic status (SES) indicators (e.g., proportion not completing high school) were more strongly associated with language and cognitive outcomes in kindergarten than were factors influencing child health at birth (e.g., low birth weight) [39]. Guhn et al. found similar findings for population-level linked data in British Columbia for mental health outcomes [40]. While several birth-related factors were significantly associated with conditions such as hyperactivity and anxiety for kindergarten-age children, as well as for children up to age 15, the largest associations with these outcomes were seen with family-level poverty.

#### 3.1.1. Area-level Socioeconomic Status and Early Child Development

Given its emphasis on area-level interpretations of whole populations, the EDI has naturally spurred research interests in examining area-level SDOH in ways that capture the breadth of available socioeconomic and demographic variables, and yet also attend to the particular context of families with young children. As Kershaw and Forer [26] point out, pan-Canadian administrative data, such as the census and income tax file data, provide a treasure trove of SDOH indicators to model area-level developmental outcomes in children. However, the choice of such indicators in the neighborhood effects literature has not been sufficiently informed by considerations relating to the intersectionality of race, class, and sex for families with young children. Kershaw and Forer’s models of EDI outcomes using custom-tabulated administrative data demonstrated the usefulness of including intersectional variables (e.g., percentage of couples with female-only earners, income inequality for lone mothers) that are rarely included in other studies of neighborhood effects.

This dual analytic strategy of widening the scope of SDOH predictors being modeled while building in intersectionality concerns has been applied recently to the development of a pan-Canadian, neighborhood-level SES index [41]. This index is a composite of 10 variables taken from the census and income tax files that accounts for almost twice as much of the variance in overall pan-Canadian vulnerability rates as other existing SES indices [42]. Most of the new index’s variables are specific to families with children under age six, with some specific to single-parent families of young children. 

Having an efficient SES index tailored to the developmental outcomes of young children in Canada is crucial in order to examine SDOH–child development associations in a variety of contexts relevant to our first research question and described in the next section. For example, Webb et al. used this new SES index to examine how EDI–SES gradients vary by children’s sex [43]. They found that the gradients were steeper for boys than girls, consistently across all developmental domains and across all Canadian provinces. More generally, it is a goal of international EDI research activities to examine the patterns of associations between SDOH and child development outcomes. Understanding the extent to which similar or different mechanisms and factors may be related to child development outcomes in different contexts and subpopulations will establish a more differentiated evidence base for identifying which actionable, changeable conditions may be addressed to enhance child development and well-being [44].

#### 3.1.2. Social Gradients in Child Development

Examining SES gradients in child development has been a ubiquitous analytic approach to demonstrating the effects of social determinants and has been employed by researchers from many countries [1,45]; we describe three examples herein using the EDI to examine such associations. In Canada, using a newly developed pan-Canadian SES index and based on EDI scores from almost 300,000 kindergarten children from essentially all Canadian jurisdictions, Forer et al. found that children in the lowest SES quintile were developmentally vulnerable at 1.5 to 1.8 times the rate of those in the highest quintile, depending on the jurisdiction [41]. Ip et al., in a study of 567 preschool children in Hong Kong, found a strong EDI–SES gradient at the child and family level of analysis [46]. The family SES index was composed of variables relating to parental education, parental occupation, family income, and family material assets. In Scotland, Woolfson et al. used the EDI to study developmental vulnerability in a sample of all 1090 Primary 1 children in one Scottish school district [27]. Using the Scottish Index of Multiple Deprivation as their index of socioeconomic status, they found that children in the lowest SES quintile were at least twice as likely as those in the highest quintile to be developmentally vulnerable. 

#### 3.1.3. Contributions of Social Determinants of Health to Prediction of Risk for Later Outcomes

Due to the population-wide implementation of the index, the EDI data, when linked with other data sources, offer the opportunity to examine developmental outcomes at kindergarten in relation to later outcomes for otherwise “undiagnosed” populations—for mental health outcomes, academic outcomes, or both.

Two sets of studies, one from Canada and one from Australia, provide examples of this opportunity. Thomson et al. studied the mental health of over 35,000 kindergarten-age children in British Columbia using EDI data. The study examined the patterns of children’s emotional maturity and social competence (based on the subdomains of the EDI) and investigated the degree to which sociodemographic variables were related to these patterns [47]. Using latent profile analysis, six distinct social–emotional profile groups were found, with membership in the lowest functioning groups associated with being male, having English as a second language, and lower household income. In a subsequent study, children were followed up to age 14 using administrative health databases [48]. The latent socioemotional functioning profiles were applied once again and were found to be associated with early-onset mental health conditions. An examination of sociodemographic characteristics revealed that boys, children in households with unmarried parents, younger mothers, and those receiving subsidies were overrepresented in the lower socioemotional functioning groups.

These findings were reflected in Australian research [49,50]. In a study by Green and colleagues, four developmental profiles were identified using the EDI domains and subdomains that were hypothesized to present varied levels of risk for future development of mental health disorders [49]. The authors found that the odds of being in the risk groups were related to several SDOH (e.g., socioeconomic disadvantage, maltreatment) and non-SDOH (e.g., parental history of mental illness and criminal offending) variables. In a study by Piotrowska and colleagues [50] linking kindergarten data to educational, health, and protection records up to 11 years of age, researchers explored the context of transition from competence to vulnerability and found that only about 22% of children deemed as typically competent on the EDI transitioned to later vulnerability; 42% of those identified with a cluster of emotional vulnerabilities in kindergarten were also vulnerable later and 41% of children with cognitive vulnerabilities remained vulnerable. Demographic factors that have been shown to impact child development and mental health, such as parental mental illness, parental offending, and evidence of use of child protection services, were powerful determinants in influencing a child’s transition between developmental profiles.

#### 3.1.4. Racial Inequalities and Early Child Development

Only a few studies using EDI data examined racial inequalities in child development. Race is a complex construct to study, and is almost impossible to study in Canada, where race and ethnicity data are rarely collected. EDI results from the US point to racism as the root cause of observed racial inequalities. Halfon et al. [51], based on a sample of over 180,000 kindergarten children in the United States, found large differences in developmental vulnerability between racial groups; specifically, vulnerability on one or more domains was 32% for Black children, 26% for Latinx children, 19% for White children, and 18% for Asian children. All groups showed the familiar gradient by neighborhood income, although it was steepest for White children and least steep for Black children. Halfon et al. concluded that equity from the start was required, and “must consider the services, supports, and interventions that children and families need to promote optimal health development” (p. 1708).

### 3.2. Studying Social Determinants of Health among Special Populations of Children

Hard-to-reach, vulnerable populations tend to be under-represented in research. As Brownell and colleagues reported in 2004, children living in lower socioeconomic (SES) neighborhoods tend to be less represented in educational data than those in higher SES neighborhoods [52]. In their analyses of Grade 3 standardized test outcomes, they found that greater percentages of children from lower SES neighborhoods either did not complete the provincial standardized tests, received an exemption from writing them, or were absent during the time the test was being written [52]. Due to the population-level reach of the EDI, it has been possible to examine associations between SDOH and developmental outcomes in a number of different special populations of children. In this section, we will focus on research involving immigrant and refugee children, children with health disorders, and children who experience maltreatment or who are placed in out-of-home care. 

#### 3.2.1. Immigrant and Refugee Children

Immigrant and refugee children represent a socially, culturally, and economically diverse group, which in Canada is a growing percentage of the population. To date, the literature on child development outcomes of immigrant and refugee children tends to be sample-based and relies on parent reports, which while an important source of data on children, may not provide a representative picture, as families who do not speak the study language fluently are often excluded and there may be mistrust towards researchers. Recently, a group of Canadian researchers started examining the associations between the SDOH and developmental outcomes using EDI data linked with a range of other datasets. For example, guided by Bronfenbrenner’s bioecological model [44,53], Milbrath and Guhn [54] examined the relationship between immigrant children’s cultural background, neighborhood-level socioeconomic factors and cultural composition, and their developmental outcomes. Their study used EDI data linked with administrative immigration records and census data to examine the effects of family and neighborhood poverty, neighborhood cultural density (in terms of being similar or not to the child’s culture), and immigrant generational status on children’s developmental health at school entry among Cantonese, Mandarin, Punjabi, and Filipino children in comparison to non-immigrant, English-speaking children. In line with previous studies, they found a negative association between family and neighborhood socioeconomic disadvantage and children’s EDI scores. They also found differences in the associations between a neighborhood’s cultural diversity and children’s developmental outcomes based on neighborhood SES indicators and children’s cultural backgrounds, with Mandarin-speaking children having lower developmental outcomes in neighborhoods with greater cultural density and Punjabi-speaking children having better developmental outcomes in poorer neighborhoods with greater cultural density. 

Another Canadian study by Gagné and colleagues [55] investigated the relationships between income and literacy and numeracy trajectories from kindergarten to Grade 7 for various groups of migrant children living in the Canadian province of British Columbia. They examined the three official categories of migrant children: economic, family, and refugee categories. They found that similarly to non-migrant children, lower income was associated with lower literacy and numeracy trajectories in all but one group of migrant children. Migrant children who were in the high-achieving economic class group were less impacted by low income. Gagné et al. [55] found that parental education levels and children’s abilities in English predicted high literacy and numeracy trajectories, despite low income. 

#### 3.2.2. Children with Health Disorders

Until recently, Canada has lacked nationally representative data pertaining to social indicators of young children’s developmental health, especially for those with health disorders. The ability to link EDI data with other datasets has allowed researchers to conduct studies on children with health disorders that were not possible before, either because of non-representative samples or because of a lack of data on certain key variables. Here, we will describe some studies from Canada and Australia that have examined SDOH in kindergarten children with health disorders.

Using pan-Canadian EDI data linked to a custom,-built neighborhood-level SES index [41], Zeraatkar and colleagues [56] examined the relationships between neighborhood-level SES and developmental health in children with disabilities, as identified in the EDI. Their results showed that all developmental domains were positively correlated with neighborhood-level SES, with the strongest relationship evident in the language and cognitive development domain. This association had already been noted in typically developing children (e.g., [41]), however this was the first Canadian population-level study to examine this link in children with disabilities. Relatedly, in Australia, O’Connor and colleagues [57] found a link between neighborhood-level SES and the odds of having an established or emerging special health-care need, with children living in the most disadvantaged neighborhoods having the highest odds of having a special health-care need. 

Other studies have focused on specific health disorders, such as autism spectrum disorder [58,59,60], fetal alcohol spectrum disorder (FASD) [61,62], and unaddressed dental needs in kindergarten [63]. These studies consistently demonstrated the relationships between children’s diagnoses, health needs, and SDOH, such as indicators of socioeconomic status at the neighborhood level. 

#### 3.2.3. Child Maltreatment and Children in Care

Developmental information on children in out-of-home care or those who experience maltreatment has been hard to come by without the opportunities to link administrative data with the EDI. Studies in Australia found that more children who have been maltreated tended to be vulnerable in all domains of their development than those who were not [64,65]. Green and colleagues [64] found that children exposed to two or more types of maltreatment and those with reported maltreatment before the age of 3 years had greater odds of being vulnerable on the EDI compared to their non-maltreated peers. Similarly, for children who were reported to child services by 5 years of age, those with the highest number of reports of maltreatment had the highest odds of being vulnerable on three or more developmental domains [65]. Maltreated children placed in the care of child protection services had slightly better developmental health in three domains (physical health and well-being, language and cognitive development, and communication skills and general knowledge) compared to maltreated children not placed in care. The authors also found that children with reports of maltreatment before the age of 18 months had the highest odds of being vulnerable in at least three domains compared to those with no maltreatment. A Canadian study reported somewhat different results. In a population-based cohort of 53,477 children living in the province of Manitoba, Wall-Weiler and colleagues [66] found that children placed in out-of-home care by child protection services were more likely to be vulnerable than children not placed in care. They also examined vulnerability levels in a subcohort of children for whom one sibling was taken into care while another one was not, as well as for discordant cousins, and did not find any differences in vulnerability between the discordant siblings or cousins. The discrepancy between the findings in the Australian and Canadian studies indicates that while children who experienced maltreatment are at risk for poor developmental outcomes, it is the larger, systemic, environmental, and social factors intersecting with microsystem characteristics (e.g., family environment) that contribute to shaping children’s developmental trajectories and that require action at policy levels.

### 3.3. Using the EDI to Evaluate Programs and Interventions

#### 3.3.1. Preschool Programs

EDI data collected in countries across the globe, such as Canada, Australia, Ireland, and Ethiopia, have been used to implement and evaluate programs meant to improve children’s developmental health at school entry [16,17,18,67]. One of the most important and ubiquitous programs put in place to help support early child development is preschool. Worldwide, up to 50% of children aged 3–5 years attend preschool [68], and preschool attendance has been associated with better school readiness and academic achievement [69,70]. An Australian study of over 250,000 children showed that preschool attendance was associated with reduced odds of developmental vulnerability during children’s first year of formal schooling, as reported by teachers in the EDI. Children who attended preschool had higher scores than those who did not in all developmental domains except emotional maturity, regardless of a child’s socioeconomic status [17]. Goldfeld and colleagues’ study emphasized most specifically the importance of continued attendance. In contrast, in a study conducted in Ireland [67], socioeconomic factors were stronger predictors of child development at school entry than preschool attendance. Children attended one year of a free preschool at any time between ages 3 years and 2 months and 4 years and 7 months, and teachers used the EDI to evaluate their development in the first year of school. Although children who participated in preschool had better social and emotional skills, and to a lesser extent better cognitive and language skills, other factors such as a child’s home life and socioeconomic status had stronger effects than preschool attendance. In addition, developmental health was relatively stable over time for most children: children starting the program with higher EDI scores tended to have higher scores than their peers at the end of the program [67]. Another recent study in Mozambique evaluated the impacts of a community-based preschool program and saw increases in all EDI domains correlated with attendance [71]. Similarly to the Ireland study, children with higher initial levels showed greater academic progress in the program. The EDI has also been utilized to evaluate the effectiveness of a comprehensive preschool curriculum in Addis Ababa, Ethiopia [16]. In one of very few randomized control intervention trials that utilized the EDI as an outcome, this study assigned children to either the regular basic preschool curriculum or to a new comprehensive preschool curriculum. The authors found that children attending the comprehensive preschool curriculum scored higher on the social competence, emotional maturity, language skills, cognitive development, communication skills, and general knowledge domains of the EDI compared to their peers receiving the basic curriculum [16]. However, the recency of this study does not allow consideration of whether this effect lasted beyond school entry.

#### 3.3.2. Early Interventions

In addition to preschool evaluations, the EDI has also been used to explore the impacts of early child development programs. One such program is the *Primeira Infância Melhor* (Better Early Childhood), a home-visiting program held in Rio Grande do Sul State, Brazil, involving regularly scheduled visits to pregnant women in their home, which continue after the child is born. The goal of this program was to help women promote their child’s health and holistic development. The EDI was used to assess the efficacy of this program. The results showed that the earlier a child exited the program, the more vulnerable they were in all five developmental domains of the EDI [19], suggesting the program was effective at improving children’s developmental health; however, a multivariate analysis found no overall difference among the study groups in terms of EDI outcomes. Many other countries have attempted to use early at-home interventions to improve developmental outcomes for marginalized communities before entering preschool [18,72]. A study by Enns et al. [18] focused specifically on the Families First Home Visiting (FFHV) program available to indigenous populations in Manitoba. An analysis of data for over 4000 families showed no significant difference in a child’s likelihood of being vulnerable in one or more domains of the EDI in comparison to non-participants. Another study of early intervention was conducted in Australia and explored the efficacy of a nurse home visiting (NHV) program, in which mothers from disadvantaged populations received home visits by a registered nurse during the immediate postnatal period. The children enrolled in this program were followed up at age five and did not show any improvement in EDI scores in comparison to children who were not involved in the NHV program [72]. 

## 4. Discussion

In this narrative review, we integrated insights from select studies that allowed us to examine the ways in which the population-based EDI data have been useful for exploring the questions raised by the Special Issue theme; that is, the extent to which population-based measurements can inform evidence-based policy in support of enhancing children’s optimal functioning [73]. In this regard, our review highlighted several points. Importantly, the population-level collection of EDI data in numerous jurisdictions internationally has provided unique opportunities to systematically examine the variability in child development outcomes in relation to social determinants of health, and to do so for subpopulations that are commonly either unrepresented or under-represented in sample-based research. The EDI has helped investigators widen the scope of research relating to the social determinants of health by virtue of its comprehensive breadth, both conceptually and analytically, in addition to as a result of the census-like nature of the data collected. The EDI also offers researchers and policy-makers the opportunity to address systemic differences in children’s development. The studies investigating the impacts of early programs and interventions using the EDI have shown inconsistent results. These inconsistences suggest that these interventions and programs may be ineffective for these children or for the domains measured with the EDI, or that the impacts of the program might be evident only in the long term. 

Studies utilizing the EDI have contributed to our understanding of the role of social context factors at multiple ecological levels (e.g., community, family) in the early development of a child. By linking the EDI with administrative data, researchers have been able to examine associations between children’s developmental health and the social determinants of health at the population level, which were previously difficult to examine. This type of research has allowed us to gain a better understanding of the socioeconomic disparities across various jurisdictions, such as with the work conducted by Forer and colleagues [41]. The EDI has also facilitated the monitoring of child developmental trajectories over time, which combined with other indicators, can inform future research and child-related policies about early developmental outcomes and predictors of later health and development.

Another advantage of the population-level data collected using the EDI is that researchers are able to study special populations of children, for whom numbers are typically low in sample-based research. Using the EDI, researchers have been able to examine the developmental health of children with autism spectrum disorder, fetal alcohol spectrum disorder, unaddressed dental needs, and children with disabilities in relation to social determinants of health. These studies have consistently demonstrated an association between neighborhood-level socioeconomic status and children’s developmental health. These studies have also been able to identify jurisdictional differences in either the prevalence of a given disorder or developmental vulnerabilities in these children. This research is vital for policy-makers, as it offers information that can help improve our ability to identify children earlier in order to provide early intervention and access to services. Some of this work is already being translated into policy briefs and recommendations (e.g., [74]). 

The EDI has also been used to evaluate early programs and interventions meant to improve children’s developmental health. Our review indicates that the results are mixed, with some studies showing a large effect (e.g., [17]), small effect (e.g., [19]), or no effect (e.g. [72]). The research examining the impacts of home visiting in particular has not shown advantages for child development at school entry. There are many potential possibilities, not the least of which is that home visiting rarely leads to overall better cognitive or behavioral outcomes in children [75]. The impacts of participating in preschool in the year prior to school entry also showed mixed results. While conceptually a sound strategy, such an intervention may not be enough to deflect the strong influence of other early social determinants, such as socioeconomic status. These studies add not only to our understanding of the limited reach of the early interventions and short preschool programs, but also to the methodological considerations in terms of evaluating their outcomes. A recent meta-analysis of early parenting interventions with a specific focus on reducing children’s disruptive behavior failed to show any evidence to support the argument for the better effectiveness of programs implemented for younger rather than older children, even though they were mostly effective [76]. 

This also gives us an opportunity to focus on the EDI’s characteristics as an instrument that provides evidence suitable for policy-level use. The EDI detects variability in early child development outcomes in a population; population-level monitoring may be the best way of capturing and examining how social determinants of health and macrosystem factors (such as implementation of preschool, variability in poverty and income, or minority status) are related to early childhood outcomes and early child development trajectories. Population-level developmental health monitoring may, thus, be an ideal tool providing evidence of the extent to which policies that significantly affect SDOH and macrosystem factors achieve lasting positive effects on developmental health outcomes, and whether such policies help to reduce inequities that exist in our societies.

Overall, results from the studies discussed in this paper show that the social determinants of health show a strong association with children’s developmental outcomes at an early age, and that the SDOH have a much stronger association than child characteristics. These findings also suggest that program interventions alone, such as preschool or home visiting, will often not be enough to compensate for the detrimental effects of poor SDOH on children’s development without addressing the more fundamental social determinants, such as poverty. 

### Limitations and Future Opportunities

It is important to acknowledge several limitations. One limitation relates to the authors’ personal preferences, which could have influenced the selection of studies reviewed in this paper. In some cases, several papers addressed the same or similar issues, and the final selection could have been swayed by the authors’ own research interests or unconscious preferences for a certain methodology, despite a thorough review of the final included papers and all authors’ consensus. All papers using the EDI are listed on the EDI bibliography website, which is constantly updated and may be easily reviewed by readers. We have not included in this review research including indigenous children, since our author team did not include indigenous members. We recognize that this is a limitation and aspire to rectify this in future reviews. Finally, the focus of this review has been on potentially unique contributions of population-based measurements of child development to inform policy-making in order to enhance children’s optimal functioning. However, a population-based lens is not a substitute for in-depth, developmental, longitudinal child development studies or evaluation studies of early interventions; rather, the different disciplinary lenses ideally complement and inform each other. We anticipate that future developmental research that draws from population-level data linkages may be able to integrate population-level data on child development at different stages of the life course—and eventually follow child cohorts intergenerationally—involving comprehensive data on children’s social context (e.g., family, community, school, socioeconomic factors, policy) and also measured during different life periods (e.g., childhood, youth, adulthood). Such comprehensive socioecological, developmental, population-based monitoring and data linkages would realize the type of developmental science that has been proposed by Urie Bronfenbrenner, in his influential formulation of the “bioecologial model of human development”. In fact, this trend is already noticeable in the EDI literature, as the papers using individual-level linkages between EDI and other data sources constitute 59% of articles published within the past five years.

## 5. Conclusions

One of the major characteristics of the EDI that lends itself well towards the population-wide studies reviewed in this paper is its holistic nature. Research demonstrates that the EDI is an effective tool for monitoring children’s developmental health, both in typically developing children and those with health disorders. Thus far, the research using the EDI has contributed to the expansion of our knowledge on the associations between SDOH and children’s developmental health, and mostly through linkages with other databases has opened many possibilities for further investigation of early childhood development. 

## Data Availability

No new data were created or analyzed in this study. Data sharing is not applicable to this article.
